# Integral assistance process implantation for ST-elevated acute coronary syndrome

**DOI:** 10.1186/cc10788

**Published:** 2012-03-20

**Authors:** JC Rodriguez-Yañez, M Celaya-Lopez, MJ Huertos-Ranchal, I Diaz-Torres, C Navarro-Ramirez, J Gomez-Ramos

**Affiliations:** 1 Hospital Universitario Puerto Real, Spain

## Introduction

The objective was to evaluate the implantation of assistance process implantation (PAI) for ST-elevated acute coronary syndrome (SCASTE) in our sanitary district. When we refer to PAI, we mean protocolysed assistance guidelines developed and published by Andalucia sanitary authorities that include recommendations to direct the assistance from the beginning of the process until patient discharge from the hospital.

## Methods

All ICU patients from HUPR diagnosed with SCASTE within the first 24 hours from January 2005 to December 2010 were included in this study and registered in the ARIAM-Andalucia Project. This database gathers the whole PAI from preadmission (PH), ER, ICU, hemodynamics laboratory and cardiology ward to discharge. Within these 6 years three main interventions were carried out: fibrinolysis protocol PH with ER and critical care unit EMS involving the ICU, continuous update of protocols based on AHA clinical guidelines, and 24-hour availability of the hemodynamic laboratory for primary coronary intervention (P-ICP available since 1 February 2007). Revascularization indexes are analyzed and grouped in 2-year periods (A, B, C), the time justified as necessary for modification after the intervention, attention times and PH action. The latter was measured by a score (aspirin, nitroglycerine, ECG, vein access, intravenous treatment and monitoring during transport) up to 6 points. A correct intervention must obtain at least 4 points. Statistical processing was by the R-UCA pack from R-Commander.

## Results

A total of 590 patients were included in this study: 188 (A), 227 (B) and 175 (C). All groups were similar in mean age, gender, IAM location and origin. A statistically significant increase was found in the revascularization and PHA attention between periods A versus C and B versus C with *P *< 0.0001 and CI (0.15 to 0.42)/(0.17 to 0.45) and (0.2 to 0.6)/(0.11 to 0.39). No statistically significant difference was found among groups A versus B. No significant difference was observed in attention times.

## Conclusion

Coordination of the SCASTE attention, constant analysis by continuous registry of different action levels (ARIAM-Andalucia registry), clinical guideline updates and adjustment to resources and environment, in this case a rural setting, meaning quality and a continuous improvement circle, reduce variability and lead undoubtedly to better assistance for our patients.

